# Older Adult (65+) Depression Prevalence increasing in New England: Findings from the 2025 Healthy Aging Data Reports

**DOI:** 10.1093/geroni/igaf122.3054

**Published:** 2025-12-31

**Authors:** Qian Song, Taylor Jansen, Tiffany Tang, Shan Qu, Yan-Jhu Su, Elizabeth Dugan

**Affiliations:** University of Massachusetts Boston, Boston, Massachusetts, United States; University of Massachusetts Boston, Boston, Massachusetts, United States; University of Massachusetts Boston, Boston, Massachusetts, United States; University of Massachusetts Boston, Boston, Massachusetts, United States; The Pennsylvania State University, University Park, Pennsylvania, United States; University of Massachusetts Boston, Boston, Massachusetts, United States

## Abstract

In 2023, the Surgeon General declared an “epidemic of loneliness” as a poll reported about one in three older adults’ experiences feeling socially isolated. The Healthy Aging Data Reports (HADR) (www.healthyagingdatareports.org) are a rich data source to study rates of older adult rates of depression. The HADR calculates the prevalence of 38 chronic diseases for each community in a state using Medicare Beneficiary Summary File (MBSF), representing 100% of traditional Medicare beneficiaries in a state, aggregating the prevalence to the community and neighborhood-levels using small area estimation techniques. The present descriptive study will compare community and neighborhood-level 65+ “ever-diagnosed” prevalence of depression over three time points (Time 1: 2014-2015; Time 2: 2017-2018; Time 3: 2020-2021) in five New England states: Connecticut (CT), Massachusetts (MA), Maine (ME), New Hampshire (NH), and Rhode Island (RI). Over the three time points, 65+ depression increased for all states. CT (T1:27.75%; T2:29.32%; T3:31.63%), MA (T1:29.52%; T2:31.2%; T3:33.02%), ME (T1:32.50%; T2:33.48%; T3:33.83%), NH (T1:26.84%; T2:27.82%; T3:28.7%), and RI (T1:31.16%; T2:31.7%; T3: 33.37%). While rates increased overtime for all states, CT reported the largest increase from time 1 to time 3 (3.9%). NH had the lowest 65+ depression prevalence in time 3 (28.7%), and Maine reported the highest (33.83%). These trends demonstrate that depression among older adults was highest during the COVID-19 pandemic (Time 3: 2020-2021) and are continuing to increase. The 2025 HADRs can be used to direct policy efforts to treat and address the growing mental health needs of the aging population.

